# Rethinking “asthma” in adult-onset “asthma” with periocular xanthogranuloma

**DOI:** 10.1016/j.jdcr.2025.11.004

**Published:** 2025-11-13

**Authors:** Alan Y. Xu, Vivian Y. Liu, Kiran Motaparthi

**Affiliations:** Department of Dermatology, University of Florida, Gainesville

**Keywords:** AAPOX, adult-onset asthma, endobronchial biopsy, non-Langerhans cell histiocytosis, orbital xanthogranulomatous disease, periocular xanthogranuloma

## Introduction

Adult orbital xanthogranulomatous disease (AOXGD) is a group of non-Langerhans cell histiocytoses with 4 clinical subtypes: adult-onset xanthogranuloma (AOX), adult-onset asthma with periocular xanthogranuloma (AAPOX), Erdheim-Chester disease (ECD), and necrobiotic xanthogranuloma (NXG).^1^ These disorders share histopathologic features including foamy histiocytes and Touton giant cells, often with infiltration of the orbicularis muscles and orbital tissue.^1^^,^^2^ However, they differ in systemic associations, radiologic features, genetic driver mutations, and prognosis. Due to their rarity, pathogenesis and optimal treatments are not well defined.

AAPOX is characterized by periocular lesions in addition to adult-onset respiratory disease.^1^^,^^3^ We describe a patient with AAPOX with similar historical, clinical, and pathologic features compared to prior descriptions of this rare disease. However, more detailed evaluation in this patient challenges the notion that respiratory disease in AAPOX truly represents asthma.

## Case presentation

**Fig 1:**
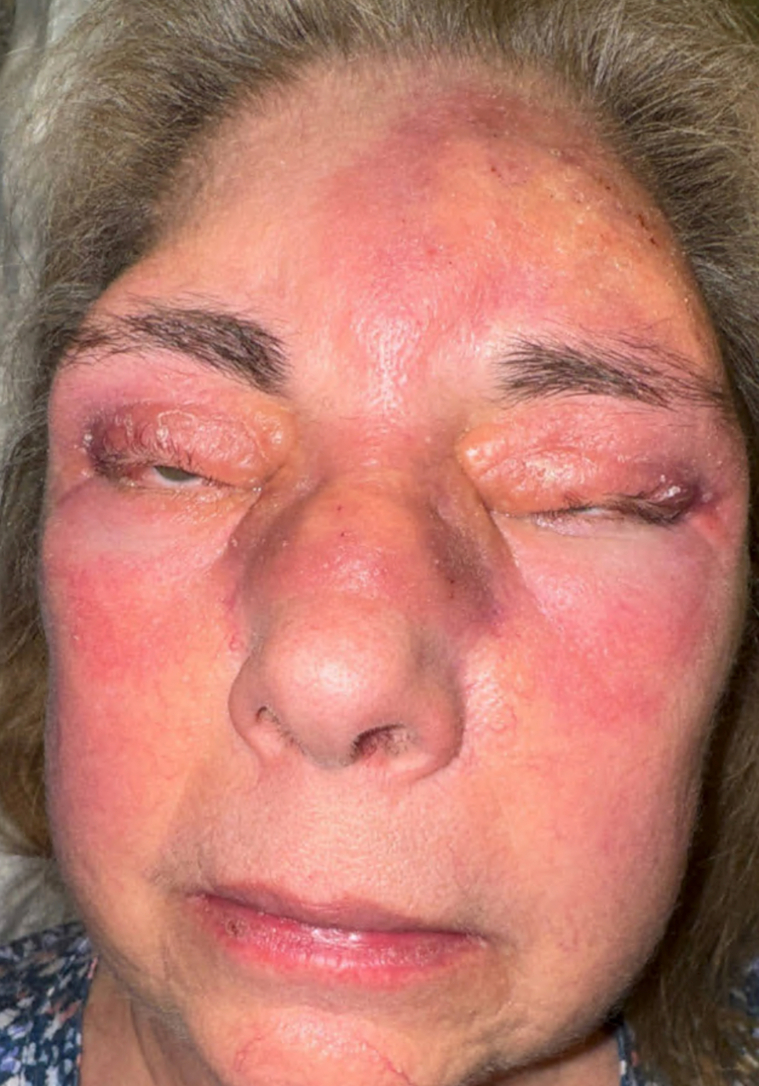
AAPOX. *Yellow*, edematous plaques on periocular skin, forehead, nose, upper cheeks.

The patient is a woman in her 60s who developed yellow periocular plaques and sinusitis symptoms (Fig 1); months later she developed symptoms resembling asthma for the first time. She had been on prednisone for almost 1 year with no improvement. She also reported headaches, dry eyes, increased thirst, and hip pain. She denied bone pain, frequent urination, or visual disturbances.

**Fig 2:**
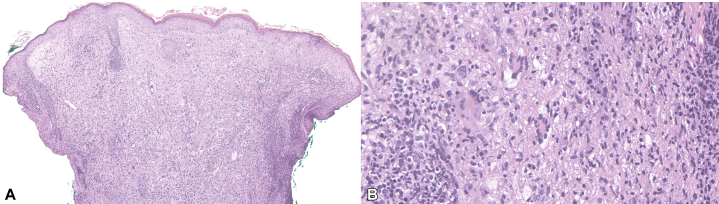
AAPOX: Dense infiltrate composed of predominantly of mononuclear foam (lipidized) histiocytes spans the dermis (**A,** H&E, 100× magnification). Multiple Touton giant cells are observed, adjacent to lymphoplasmacytic aggregates (**B,** H&E, 200× magnification).

Skin biopsies showed mononuclear foamy histiocytes with scattered Touton giant cells and dense lymphoplasmacytic aggregates (Fig 2). Up to 50 IgG4-positive plasma cells were identified per high-power field, representing up to 40% of the IgG-positive plasma cells. Serum IgG4, serum protein electrophoresis and flow cytometry were unremarkable. Maxillofacial computed tomography (CT) revealed soft tissue fullness involving the periorbital and supraorbital regions, scalp, anterior nasal septum, lacrimal glands, palatine tonsils, nasopharynx, parapharyngeal space, and nasal cavity. Magnetic resonance imaging (MRI) of the brain demonstrated an infiltrative process involving the face, orbits, nasal cavities, nasopharynx, and scalp, with intracranial extension involving the dura and middle and anterior cranial fossae. There was also nasal septal perforation and complete opacification of the sphenoid sinuses. Chest CT revealed circumferential tracheal wall thickening extending into the bronchi, prominent mediastinal lymph nodes, and pulmonary nodules. A recent echocardiogram was unremarkable. Bone radiographs and bone scintigraphy showed no evidence of osteosclerosis—a hallmark of ECD with 95% sensitivity.^4^ Consultation with endocrinology showed no evidence of diabetes insipidus. She was also evaluated by pulmonology; pulmonary function testing was normal, and imaging findings were not consistent with asthma. Biopsies obtained from both the nasal and endobronchial mucosae mirrored skin findings, revealing foamy histiocytes. The lung tissue was positive for cyclin D1 and negative for S-100, CD1a, BRAFV600E, and ALK. Next-generation sequencing with a total of 324 genes sequenced revealed no genomic alterations associated with histiocytosis. Overall, the clinical findings, pathology, and imaging supported a diagnosis of AAPOX and allowed distinction from the other clinical subtypes of AOXGD.

The patient’s respiratory disease was previously refractory to antihistamines, montelukast, and prednisone. Symptoms of sinusitis persisted despite multiple sinuplasty procedures. Tocilizumab and subcutaneous immune globulin were also ineffective, and the cutaneous and pulmonary findings were refractory to chronic high-dose prednisone. Prednisone was tapered and tocilizumab was discontinued. Methotrexate 15 mg weekly was initiated, along with rituximab 1 g IV on days 0 and 14, with subsequent improvement in the periocular plaques and respiratory symptoms. At 6 months follow-up, she remained on maintenance rituximab dosing, while methotrexate was discontinued. Residual lesions over the nasal dorsum and forehead responded to radiotherapy. Her cough markedly improved, with no dyspnea.

## Discussion

Prior descriptions of AAPOX include respiratory disease described as adult-onset asthma.^2^^,^^3^^,^^5^ The pathogenesis of asthma-like symptoms in the setting of AAPOX, however, is unclear. Some authors believe that AAPOX develops from an atopic etiology, as it is associated with chronic rhinosinusitis, nasal polyps, elevated IgE, and eosinophilic infiltration of periorbital tissue.^1^, ^2^, ^3^^,^^6^ Alternatively, the accumulation of histiocytes in periorbital tissue may be linked to activation of the mononuclear phagocytic system via dendritic cells in the airway epithelium and submucosa.^2^ Overlapping features of IgG4-related disease—lymphoplasmacytic infiltrates with IgG4-positive plasma cells, involvement of the orbits and lungs, and response to rituximab—are also increasingly recognized in AAPOX. However, the pathologic findings confirming a histiocytosis, the absence of fibrosis and obliterative phlebitis, and the normal serum IgG4 level excluded IgG4-related disease in this patient.^7^

A review of the literature found that routine pulmonary work-up and monitoring are lacking (Table I). When obtained, CT scans demonstrated hilar lymph node enlargement, reticulonodular infiltrates, peribronchial thickening, small subpleural, interstitial, and fissural pulmonary nodules, and bronchiolitis-like opacities. PET CT scans also showed axillary and mediastinal lymphadenopathy.^6^ Chest x-rays were usually normal.^3^ Pulmonary function tests typically showed an obstructive pattern.^2^^,^^3^^,^^8^ However, nasal or pulmonary biopsies were not described in the literature to date. In our patient, the endobronchial biopsy findings and questionable features of asthma suggest that the asthma-like symptoms of AAPOX reflect histiocytic infiltration of the respiratory tract that mirrors the cutaneous disease.

**Table I:** Review of pulmonary findings, treatment, and disease response described in prior case reports and series of AAPOX

Pulmonary symptoms	Chest imaging	Treatment	Response to treatment	Source
Asthma	Small interstitial nodules and hilar lymph node enlargement	Oral methylprednisolone and methotrexate	Not mentioned	Sahu et al, 2020
Asthma	Not mentioned	Intralesional triamcinolone acetonide (eyelids)	Not mentioned	Knani et al, 2023
Asthma	Not mentioned	Prednisolone; debulking; methotrexate; azathioprine; mycophenolic acid	Not mentioned	Detiger et al, 2022
Asthma; allergic sinusitis	Hypermetabolic mediastinal lymph nodes	Oral prednisone; methotrexate	Asthma resolved	London et al, 2015
Not mentioned	Not mentioned	Prednisolone; rituximab	Clinical and imaging signs subsided significantly	Asproudis et al, 2019
Asthma	Generalized lymphadenopathy of axillary and mediastinal lymph nodes	Rituximab	Not mentioned	Sahu et al, 2025
Asthma	Not mentioned	Surgery, corticosteroids, chemotherapy, radiation	Not mentioned	Sivak-Callcott et al, 2006
Asthma	Not mentioned	Intralesional triamcinolone; anterior orbitotomy; rituximab	Not mentioned	Barke et al, 2023
Asthma	Not mentioned	Anterior orbitotomy	Not mentioned	Santos et al, 2022
Asthma	Not mentioned	Methylprednisolone; rituximab	Not mentioned	Burris et al, 2016
Asthma; rhinosinusitis	Nodular ground-glass opacities, considered reactive	Prednisone; methotextrate	Substantially improved symptoms of rhinosinusitis and asthma	Chhabra et al, 2022
Asthma	Normal	Debulking; orbitotomy; intralesional triamcinolone acetonide	Not mentioned	Fekri et al, 2024
Asthma	Not mentioned	Prednisone; cyclophosphamide; intralesional triamcinolone; orbitotomy; debulking	Not mentioned	Green et al, 2021

*AAPOX*, Adult-onset asthma with periocular xanthogranuloma.

Overall, treatments for AAPOX have shown variable efficacy. Improvements in eyelid swelling were more likely to occur than improvements in the yellow plaques, while symptoms of asthma showed variable response to the various treatments. In general, systemic corticosteroids led to improvement in periocular swelling and asthma but with high recurrence after tapering.^2^^,^^3^^,^^6^^,^^8^ Steroid-sparing agents including methotrexate, cyclophosphamide, and azathioprine were used with variable response.^2^^,^^6^^,^^9^ Intralesional triamcinolone, orbitotomy and radiotherapy showed varied success in periocular lesions. Finally, some patients have responded well to rituximab, presumably due to the presence of CD20-positive B cells within the dense lymphoplasmacytic aggregates observed in biopsies of AAPOX.^9^ Furthermore, the MAPK pathway is targeted in all subtypes of AOXGD, providing the potential for targeted therapy using BRAF or MEK inhibitors in the treatment of AAPOX and other clinical subtypes. However, mutations in the MAPK pathway may be difficult to detect with next-generation sequencing, given that the true proportion of mutation-bearing histiocytes in AOXGD is low.^10^

## Conclusion

The asthma-like symptoms seen in AAPOX likely represent underlying histiocytic infiltration of the respiratory tract rather than true asthma. Treatment of AAPOX is difficult, as cutaneous findings and respiratory disease often do not fully respond. Surrogate immunohistochemistry, next-generation sequencing, or digital droplet PCR are necessary to determine the applicability of targeted therapies.

## Conflicts of interest

None disclosed.
